# Assessing the Influence of Inorganic Nanoparticles on the Mechanical and Tribological Performance of PPS-Based Composites: A Comparative Study

**DOI:** 10.3390/polym17192573

**Published:** 2025-09-23

**Authors:** Jixiang Li, Mei Liang, Xiaowen Zhao, Shengtai Zhou, Huawei Zou

**Affiliations:** State Key Laboratory of Advanced Polymer Materials, Polymer Research Institute, Sichuan University, Chengdu 610065, China; jixiangmr@163.com (J.L.); liangmeiww@163.com (M.L.); zhaoxiaowenscu@126.com (X.Z.)

**Keywords:** polyphenylene sulphide, γ-irradiated poly(tetrafluoroethylene), inorganic nanoparticles, tribological properties

## Abstract

In this work, γ-irradiated poly(tetrafluoroethylene) (i-PTFE) and short carbon fibre (SCF) along with different types of ceramic inorganic nanoparticles (i.e., SiC, SiO_2_, ZnO, TiO_2_, and CaCO_3_) were employed to improve the mechanical and tribological performance of polyphenylene sulphide (PPS) composites. The results showed that the flexural strength and modulus of PPS composites increased with the addition of inorganic nanoparticles. Moreover, the inorganic nanoparticles not only exhibited a ‘micro-bearing’ effect during friction tests, but also promoted the formation of high-quality transfer film on the surface of a friction pair, significantly improving the self-lubricating performance of PPS composites. XPS analysis confirmed the occurrence of friction-induced chemical reactions during the friction process in nanoparticle-containing PPS/i-PTFE/SCF composites, which was helpful in improving the tribological performance. PPS/i-PTFE/SCF/SiC composite demonstrated an average friction coefficient of 0.083 and specific wear rate of 9.04 × 10^−6^ mm^3^/Nm, which was the best among the studied systems. This work provided valuable insights for developing high-performance self-lubricating polymer composites that can be applied in high-end engineering sectors.

## 1. Introduction

Friction plays an important role in power transmission and control; however, the related energy loss, material wear, and temperature rise increase the cost of maintenance and risk of malfunction [1,2,3,4]. According to reports, approximately 1/3 of global energy is consumed in the form of friction, and approximately 80% of mechanical failures in industrial sectors is caused by abrasion-induced component failure [5,6]. Self-lubricating polymer composites, which are characteristic of lightweightness, ease of melt processing, and flexible designability, are garnering attention in both academic and industrial spheres to ameliorate friction-induced wear loss in the fields of transportation, machinery equipment, biomedicine, and electronics fields among others [7,8,9].

Polyphenylene sulphide (PPS), as a high-performance engineering thermoplastic polymer, is widely used in the automotive, electronics, and aviation industries [10,11]. However, due to its poor friction and wear performance, the application of PPS in the field of mechanical engineering is limited [12,13]. According to a previous report, the average friction coefficient and specific wear rate of PPS reached as high as 0.564 and 3.55 × 10^−3^ mm^3^/Nm, respectively [14]. Thus, it becomes imperative to improve the tribological performance while maintaining excellent mechanical properties of PPS to broaden its application in high-end engineering sectors.

In recent years, there has been a surge in modifying the tribological performance of PPS, with the main methods including polymer blending, solid lubricant addition, fibre and fabric reinforcement, nanoparticle modification, and multi-component compound modification [15,16,17,18,19,20]. For example, Kim et al. [15] studied the friction and wear behaviour of PPS by blending ethylene-butyl acrylate (EBA). The results showed that the friction coefficient and wear rate of PPS/EBA blends decreased with increasing content of EBA. In addition, the wear mechanism of PPS changed from adhesive wear to abrasive wear with the presence of EBA.

Solid lubricants, including graphite (Gr), boron nitride (BN), polytetrafluoroethylene (PTFE), molybdenum disulphide (MoS_2_), etc., are able to separate the polymer matrix from the surface of the friction pair surface by promoting the formation of intact transfer film, thus improving the tribological performance [21]. Yu et al. [16] studied the influence of different types of solid lubricants (Gr, MoS_2_, or PTFE) on the properties of PPS composites. They found that the introduction of PTFE greatly improved the wear resistance of PPS, while MoS_2_ had a negative effect. In addition, the presence of PTFE promoted the occurrence of friction-induced chemical reactions to generate compounds such as FeS and FeSO_4_ that helped to improve the adhesive strength of transfer film. However, PTFE is highly inert and is hard to achieve uniform dispersion with in polymer composite, resulting in limited improvement in terms of tribological performance [22]. Therefore, several surface modification methods for PTFE were reported, including chemical etching [23,24], plasma treatment [25], and radiation irradiation treatment [26,27].

In a previous work, the modification of PTFE by γ-irradiation treatment resulted in the formation of a certain amount of polar groups on the surface of PTFE, which facilitated the distribution of PTFE particles in the substrate and enhanced the tribological modification effect [28]. Zhao et al. [29] conducted research on assessing the tribological properties of PPS composites which were modified by Ag_2_S and PbTe particles. The results showed that Ag_2_S underwent friction-induced chemical reaction during the sliding process, promoting the formation of a uniform and adhesive transfer film that favoured the reduction of friction and wear properties. However, the presence of PbTe particles demonstrated adverse effect which exacerbated the abrasion of PPS substrate. Yu et al. [30] studied the influence of CuS, CaF_2_, and ZnF_2_ particles on the friction and wear behaviour of PPS. Their results showed that CuS had significantly improved the wear resistance of PPS, while CaF_2_ and ZnF_2_ exhibited a negative effect. Furthermore, they also investigated the effect of Al_2_O_3_, SiC, Si_3_N_4_, and Cr_3_C_2_ particles on the tribological properties of PPS. The results revealed that, except for Al_2_O_3_, the other particles significantly improved the wear resistance. XPS analysis indicated that the inorganic particles promoted the partial decomposition of PPS during the friction process and generated chemical compounds that were beneficial for improving wear resistance. However, in the other scenarios, the inorganic particles acted as solid abrasives amid the surface of the friction pair and sample block, which damaged the transfer film and exacerbated wear loss [31].

The above studies demonstrated that there are significant differences of inorganic particles on modifying the tribological performance of PPS, and the specific mechanism remains unclear. Cho et al. [32] studied the effect of CuO nanoparticles, carbon fibres (CF), and Kevlar fibres on the friction and wear behaviour of PPS. The results showed that the simultaneous addition of CuO and CF synergistically improved the tribological performance when compared with single filler modification. Due to the lubricating effect of carbon, the friction coefficient of PPS/CF composite was half of the PPS/Kevlar counterpart. Aslan et al. [33] investigated the modification effects of hexagonal boron nitride (hBN) and/or graphene nanosheets (GnP) on basalt fibre (BF)-reinforced PPS composites. The results indicated that the combined addition of hBN and GnP in PPS/BF composite provided a synergistic lubrication effect and constructed heat conduction pathways, which were conducive to reducing the accumulation of friction-induced heat, thereby playing a role in reducing the friction and wear. However, these studies lack a thorough examination of the tribological mechanisms. Furthermore, the neglect of mechanical properties in previous research prevents the application of self-lubricating polymer composites as structural components in engineering fields. In the present work, γ-irradiated PTFE (i-PTFE) and short carbon fibres (SCF) along with different types of inorganic nanoparticles (such as SiC, SiO_2_, ZnO, TiO_2_, or CaCO_3_) were employed as solid additives to improve the tribological performance of PPS-based composites. This work focused on the influence of inorganic filler type on the performance of PPS-based composites, aiming to elucidate the mechanism that related to the overall improvement of both mechanical and tribological properties. This work provided a facile strategy to fabricate high-performance self-lubricating polymer composites that can be applied in engineering sectors such as aerospace, automotive, and machinery among others.

## 2. Materials and Methods

### 2.1. Materials

Polyphenylene sulphide (PPS) was purchased from Deyang Kejia High-Tech Material Co., Ltd., (Deyang, China). γ-irradiated polytetrafluoroethylene (i-PTFE) with an average particle size of 200–300 nm was purchased from Sichuan Golden Nuclear Irradiation Technology Co., Ltd., (Meishan, China). Polyacrylonitrile-based short carbon fibres (SCF), with an average length of 3 mm and a diameter of 7 μm, were provided by Shanghai Lishuo Composite Material Technology Co., Ltd., (Shanghai, China). Silicon carbide (SiC) particles with an average dimeter of 30 nm, silicon dioxide (SiO_2_) particles with an average diameter of 50 nm, zinc oxide (ZnO) particles with an average diameter of 20 nm, titanium dioxide (TiO_2_) with an average diameter of 20 nm, and calcium carbonate (CaCO_3_) with an average diameter of 20 nm were purchased from Zhejiang Yamei Nanotechnology Co., Ltd., (Jiaxing, China).

### 2.2. Fabrication of PPS Composites

Prior to melt blending, all materials were dried at 80 °C for 12 h. Then, raw materials were added to a co-rotating twin-screw extruder (TSSJ-25/33, Chengdu Tarise Chemical Engineering Co, Ltd., Chengdu, China) in accordance with the formulations specified in Table 1. The temperatures of different heating zones from the hopper to extrusion die were set at 230, 275, 285, 285, 290, 290, 290, 285, and 285 °C, respectively. The diameter of the screws was 25 mm with an aspect ratio of 33. The screw rotation speed was 280 rpm and the feed rate was about 1.35 kg/h. The specimens for testing were prepared using a servo injection moulding machine (MA2000, Ningbo Haitian Plastic Machinery Group Co., Ltd., Ningbo, China). The temperatures of different heating zones were set at 270, 285, 295, 295, and 300 °C, respectively. The mould temperature and injection pressure were set at 100 °C and 120 MPa, respectively. The cooling time and holding pressure were 35 s and 80 MPa, respectively.

### 2.3. Characterizations

**Figure 1:**
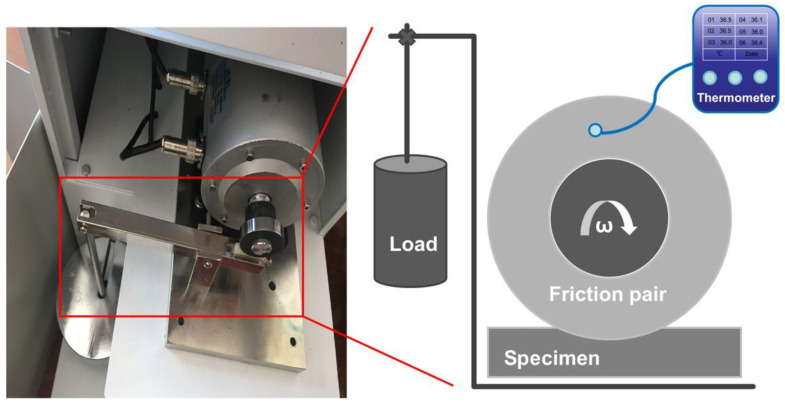
The experimental setup for conducting friction tests.

The tensile and flexural properties of samples were tested using a universal testing machine (Instron 5567, Instron, Boston, MA, USA) in accordance with GB/T 1040.2-2022 [34] and GB/T 9341-2008 [35], respectively. The surface hardness was determined in accordance with GB/T 2411-2008 [36] using a Shore D hardness tester (Yueqing Handpi Instruments Co., Ltd., Wenzhou, China). As depicted in Figure 1, the sliding friction and wear tests were conducted in accordance with GB/T 3960-2016 [37] using a block-on-ring tester (M-200A, Beijing Guance Jingdian Instrument Equipment Co., Ltd., Beijing, China) for a duration of 1 h under a nominal load of 200 N. The dimensions of the testing specimens were 30 × 7 × 6 mm^3^. The calculation of average friction coefficient (μ) and specific wear rate (δ), which are key parameters for assessing the tribological performance, is mentioned in previous studies [38,39]. The real-time temperature of the friction pair was in situ monitored using a multi-path thermometer (AT4204, Changzhou Applent Instruments Ltd., Changzhou, China) during friction tests. A Hot Disk thermal constants analyzer (TPS 2500 S, Hot Disk AB, Gothenburg, Sweden) was used to evaluate the thermal conductivity of samples in accordance with GB/T 42919.1-2023 [40]. The microstructure of samples after fracture and friction test was observed using a scanning electron microscope (JSM-9600, JEOL, Tokyo, Japan). A 3D optical profilometer (KEYENCE, VR6200, Osaka, Japan) was used to capture the feature of worn surface, including worn width (R_w_) and depth (R_d_). Elemental analysis of the surface of the metal friction pair was performed using an energy-dispersive X-ray spectrometer (Ultim Max, Oxford Instruments Co., Ltd., Oxford, UK). The surface chemical state of specimens after friction test was determined using an X-ray photoelectron spectrometer (AXIS Supra, Kratos Instruments Co., Ltd., Manchester, UK). The melting behaviour of samples was examined using differential scanning calorimetry (DSC, DSC204, NETZSCH, Schlierbach, Germany). Samples weighed 5~8 mg were heated from 30 to 310 °C at 10 °C/min under nitrogen atmosphere. The calculation of crystallinity (X_c_) was mentioned in our previous research [39].

## 3. Results and Discussions

### 3.1. Morphology

**Figure 2:**
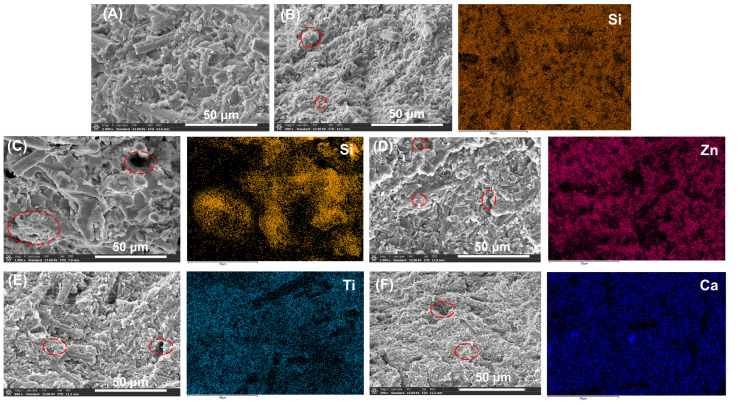
The morphology and elemental analysis of (**A**) P/i-PTFE/SCF, (**B**) P/i-PTFE/SCF/SiC, (**C**) P/i-PTFE/SCF/SiO_2_, (**D**) P/i-PTFE/SCF/ZnO, (**E**) P/i-PTFE/SCF/TiO_2_, and (**F**) P/i-PTFE/SCF/CaCO_3._

The morphology and elemental distribution of PPS-based composites was observed using SEM and EDS, as displayed in Figure 2. Figure 2A showed that CFs were randomly interwoven in P/i-PTFE/SCF composites. In comparison with Figure 2A, Figure 2B–F revealed that PPS composites with different types of inorganic nanoparticles exhibited a dense filler packing structure along with the presence of filler agglomerates (as indicated by the red contours). On one hand, the inorganic nanoparticles filled the gaps amid evenly distributed CFs, which increased the packing density of inorganic fillers [41,42]. On the other hand, the filler agglomerates acted as stress concentration points which are detrimental to mechanical performance [43]. Meanwhile, the encapsulation of CFs by PPS matrix was, to a certain extent, impaired, thus hindering the mechanical interlocking effect of CFs within the composites.

### 3.2. Mechanical Properties

**Figure 3:**
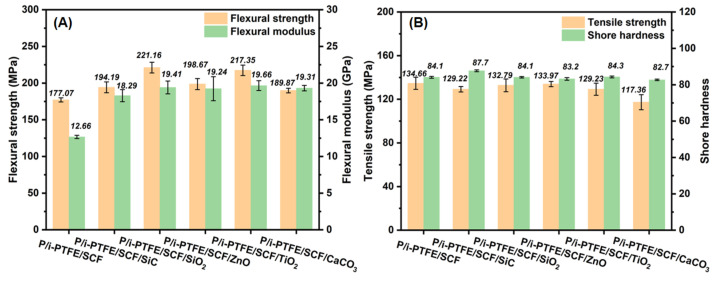
(**A**) Flexural strength and modulus; (**B**) tensile strength and surface hardness of inorganic nanoparticles-modified P/i-PTFE/SCF composites.

As shown in Figure 3A, the flexural strength and modulus of P/i-PTFE/SCF composite increased with the addition of inorganic nanoparticles. This was likely due to the fact that the presence of inorganic nanoparticles improved the packing density of fillers, which helped to increase the stiffness [44,45]. Additionally, the inorganic nanoparticles dissipated and absorbed a certain amount of energy during the mechanical bending process. However, the agglomerated nanoparticles exhibited poor interfacial bonding within the substrate and they had an adverse effect on the mechanical interlocking of CFs. Figure 3B shows that the tensile strength of P/i-PTFE/SCF composite was slightly decreased with the addition of inorganic nanoparticles. Additionally, it is worth noting that the P/i-PTFE/SCF/SiC composite exhibited higher hardness when compared with the other composites, which is primarily due to the high rigidity of SiC.

### 3.3. Thermal Properties

**Figure 4:**
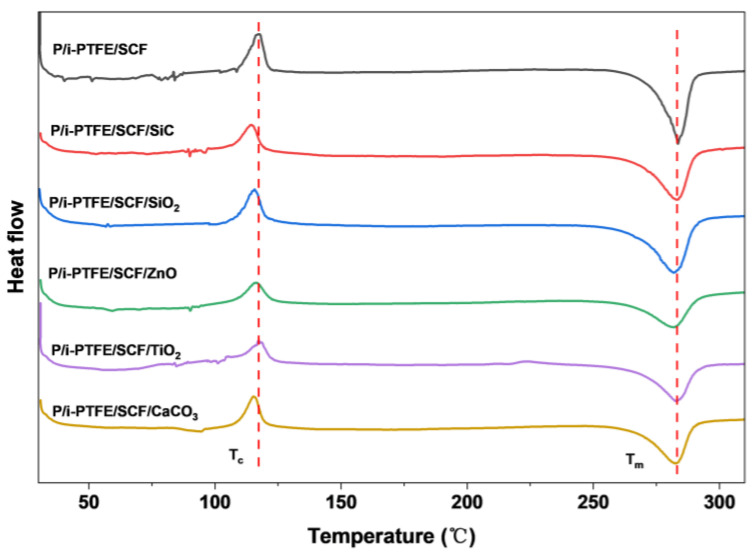
The melting behaviour of PPS-based composite with different inorganic nanoparticles.

The melting behaviour of PPS-based composites with different types of inorganic nanoparticles are displayed in Figure 4. The results showed that two distinct peaks are observed which correspond to the cold crystallisation of PPS (T_c_, approximately 117 °C) and the melting of PPS crystals (T_m_, approximately 285 °C). As shown in Figure 4, the area of the cold crystallisation peak was decreased with the introduction of inorganic nanoparticles, which was attributed to the steric hindrance effect of inorganic nanoparticles on the rearrangement of polymer chains. Moreover, the presence of inorganic nanoparticles provided heterogeneous crystallization sites that led to a decrease of the T_c_, which was particularly obvious for the SiC-containing composites. The crystallinity (X_c_) of PPS in corresponding composites is listed in Table 2. The results showed that both the melting enthalpy and X_c_ of inorganic nanoparticles-containing composites were decreased when compared with those of the P/i-PTFE/SCF counterpart. Under such circumstances, the spatial confinement effect of the inorganic nanoparticles was believed responsible for the above observations.

### 3.4. Tribological Properties

**Figure 5:**
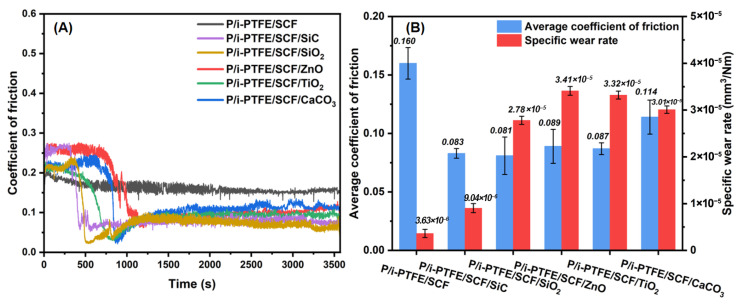
(**A**) The transient μ of different PPS composites; (**B**) the average μ and δ of different inorganic nanoparticle-containing PPS/i-PTFE/SCF composites.

As shown in Figure 5A, the stability of P/i-PTFE/SCF composites during the friction process was significantly deteriorated with the addition of inorganic nanoparticles. For example, the composites exhibited a higher μ during the running-in stage when compared with the P/i-PTFE/SCF counterpart, indicating that the nanoparticle-containing composites were subject to severe frictional force. This is likely due to the enhancement of surface roughness and stiffness by loading inorganic nanoparticles. Additionally, the exfoliation of inorganic nanoparticles acted as three-body abrasives during the friction process. As the friction process progressed, the μ of inorganic nanoparticles-modified P/i-PTFE/SCF composites first underwent a sharp decrease before entering the stable state. This may be due to the fact that the inorganic nanoparticle agglomerates were peeled off under the sliding action of the metal friction pair, which significantly reduced the friction contact area. At the same time, the presence of inorganic nanoparticles was beneficial for generating the micro-bearing effect and forming a lubricating transfer film on the surface of the metal friction pair, thereby causing a sudden drop of frictional force. Among these, the friction curve of P/i-PTFE/SCF composite with SiC or SiO_2_ showed an earlier stabilisation stage than the other nanoparticles, exhibiting a greater degree of lubrication effect.

As shown in Figure 5B, the average μ of P/i-PTFE/SCF composite decreased with the addition of inorganic nanoparticles, but the δ increased to varying degrees. This may be due to the fact that the inorganic nanoparticles promoted the formation of a lubricating transfer film during the friction process and transformed the sliding friction into rolling friction, thereby achieving a self-lubricating improvement effect and reducing μ. However, due to the poor compatibility of inorganic nanoparticles with the substrate, they acted as three-body abrasives under the continuous action of the metal friction pair, thereby resulting in severe wear loss. Among these, the composite which was modified using SiC nanoparticles exhibited the best tribological performance, which reached an average μ of 0.083 and δ of 9.04 × 10^−6^ mm^3^/Nm, respectively.

**Figure 6:**
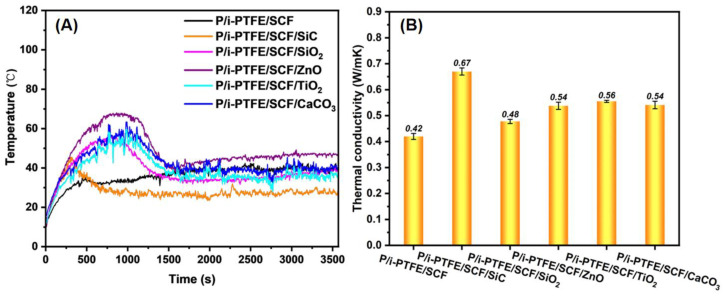
(**A**) The transient friction temperature and (**B**) thermal conductivity of inorganic nanoparticles-containing PPS/i-PTFE/SCF composites.

As shown in Figure 6A, P/i-PTFE/SCF composite experienced a rapid increase in temperature during the initial stage of the friction test. As the friction progressed, the friction temperature gradually decreased and reached a stable stage. Combining the transient μ in Figure 5A, the initial sharp increase of friction temperature was related to the severe frictional force experienced by the composite during the early stage of friction. In the later stage of the friction test, the temperature of the composite gradually decreased and stabilised, which was attributed to the following factors: (1) the rolling effect of inorganic nanoparticles and the formation of a lubricating transfer film significantly reduced the actual contact area, thus lowering frictional force; (2) the formation of efficient heat conduction pathways (as reflected by the increase of thermal conductivity in Figure 6B) by inorganic nanoparticles and SCFs favoured the heat dissipation during the friction process. This allowed the generated friction heat to dissipate in a timely manner, thereby reducing friction temperature. As a severe heat accumulation caused the temperature of friction contact surface to rise, leading to the aggravation of friction-induced wear loss. Figure 6B showed that the P/i-PTFE/SCF/SiC composites exhibited the highest thermal conductivity among the studied systems, which was in accordance with its lower δ than the other inorganic nanoparticles-containing counterparts (see Figure 5B).

### 3.5. Wear Mechanism

**Figure 7:**
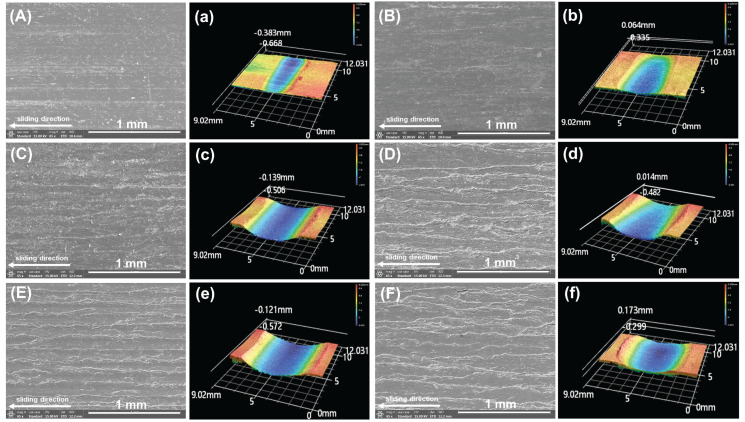
The morphology and 3D profile of the worn surface: (**A**,**a**) P/i-PTFE/SCF, (**B**,**b**) P/i-PTFE/SCF/SiC, (**C**,**c**) P/i-PTFE/SCF/SiO_2_, (**D**,**d**) P/i-PTFE/SCF/ZnO, (**E**,**e**) P/i-PTFE/SCF/TiO_2_, and (**F**,**f**) P/i-PTFE/SCF/CaCO_3_.

As shown in Figure 7A–F, the worn surface of P/i-PTFE/SCF composite became rougher after adding inorganic nanoparticles, and SEM images revealed that the worn surface exhibited obvious nanoparticle exposure and irregular plough grooves, indicating characteristics of abrasive wear. This was attributed to the limited interfacial bonding of inorganic nanoparticles within the composite. Under the continuous shear action of the metal friction pair, the nanoparticles were continuously peeled off and they acted as third-body abrasive at the friction interface. Additionally, it can be observed that the worn surface of P/i-PTFE/SCF composite with inorganic nanoparticles exhibited a significant amount of lubricating film. This was attributed to the micro-bearing effect of inorganic nanoparticles and the formation of a lubricating transfer film, which helped prevent further exacerbation of friction and wear. As shown in Figure 7b and Table 3, P/i-PTFE/SCF/SiC composite exhibited a relatively lower wear level than the other inorganic nanoparticle-containing counterparts. This was because P/i-PTFE/SCF/SiC composite quickly transited from the running-in stage to stable wear stage during the friction process, forming a uniform lubricating transfer film. Additionally, its excellent heat dissipation capability prevented the severe accumulation of friction heat at the friction interface from adversely affecting the performance of corresponding composites.

**Figure 8:**
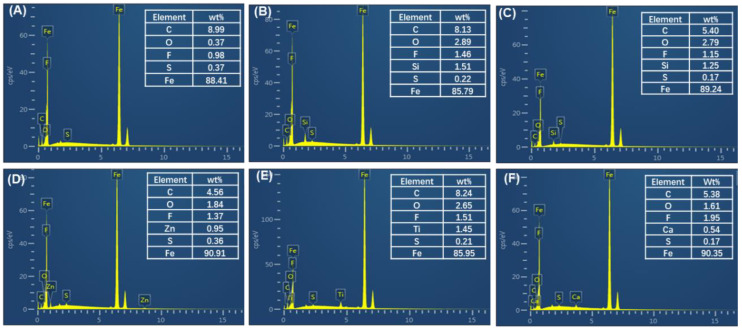
EDS elemental analysis of transfer film on the surface of the metal friction pair: (**A**) P/i-PTFE/SCF, (**B**) P/i-PTFE/SCF/SiC, (**C**) P/i-PTFE/SCF/SiO_2_, (**D**) P/i-PTFE/SCF/ZnO, (**E**) P/i-PTFE/SCF/TiO_2_, and (**F**) P/i-PTFE/SCF/CaCO_3_.

Figure 8B–F shows the elemental analysis of transfer films on the surface of metal friction pairs. The results showed that under the applied shear force of the metal friction pair, inorganic nanoparticles underwent adhesion and transfer. Additionally, compared with P/i-PTFE/SCF, the addition of inorganic nanoparticles significantly increased the content of F element in the transfer film. This phenomenon was attributed to the following reasons: (1) inorganic nanoparticles hindered the exposure of CFs within the composite, thereby reducing direct damage to the transfer film during friction tests; (2) inorganic nanoparticles enhanced the adhesion strength of i-PTFE through friction chemical reactions during the friction process. Combining with the significant reduction in μ of P/i-PTFE/SCF composite in Figure 5B, this indicated that the multi-component transfer film which was formed on the surface of the metal friction pair during the friction process exhibited excellent lubricating effects.

### 3.6. Friction-Induced Chemical Reactions

**Figure 9:**
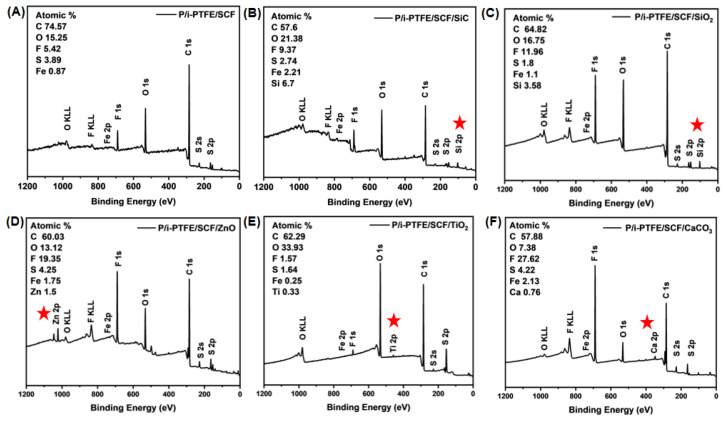
XPS full spectrum on the worn surface: (**A**) P/i-PTFE/SCF, (**B**) P/i-PTFE/SCF/SiC, (**C**) P/i-PTFE/SCF/SiO_2_, (**D**) P/i-PTFE/SCF/ZnO, (**E**) P/i-PTFE/SCF/TiO_2_, and (**F**) P/i-PTFE/SCF/CaCO_3_.

To further investigate the self-lubrication mechanism of inorganic nanoparticle-modified P/i-PTFE/SCF composites, XPS analysis was performed on the worn surfaces. As shown in Figure 9A–F, the XPS full spectra exhibited C1s, F1s, O1s, Fe2p, and S2p peaks, which originated from the friction-induced chemical reactions involving PPS, i-PTFE, SCF, the metal friction pair, O_2_, and H_2_O. In a previous work, the presence of products such as [-C_6_H_4_-S-]n, CF_2_, Fe_2_O_3_, Fe_2_(SO_4_)_3_, and C-SO_x_-C on the worn surface of PPS-based composites was confirmed [46]. Additionally, with the addition of different inorganic nanoparticles, characteristic element peaks (Si2p, Zn2p, Ti2p, and Ca2p) appeared in the full spectrum of P/i-PTFE/SCF composite (as indicated by the red star).

**Figure 10:**
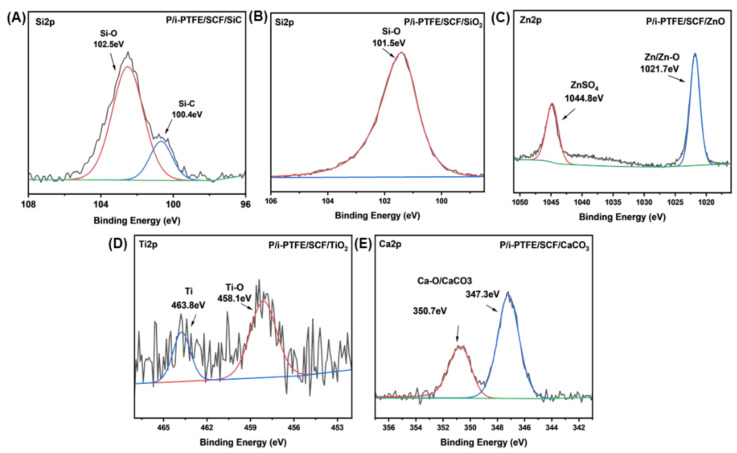
XPS energy spectrum of characteristic elements on the worn surface: (**A**) P/i-PTFE/SCF/SiC, (**B**) P/i-PTFE/SCF/SiO_2_, (**C**) P/i-PTFE/SCF/ZnO, (**D**) P/i-PTFE/SCF/TiO_2_, and (**E**) P/i-PTFE/SCF/CaCO_3_.

As shown in Figure 10A–E, the chemical state of inorganic nanoparticles was determined from the characteristic element energy spectra corresponding to each composite. It is observed that partial SiC nanoparticles in P/i-PTFE/SCF/SiC composite underwent thermal oxidation reactions, resulting in the formation of SiO_2_ (102.5 eV) [47]. The Si2p energy spectrum which corresponded to P/i-PTFE/SCF/SiO_2_ indicated that SiO_2_ particles did not undergo the friction chemical reactions. In the Zn2p energy spectrum which corresponded to P/i-PTFE/SCF/ZnO, 1021.7 and 1044.8 eV belonged to Zn/ZnO and ZnSO_4_, respectively [48]. In the Ti2p energy spectrum of P/i-PTFE/SCF/TiO_2_, 463.8 and 458.1 eV were attributed to Ti and TiO_2_, respectively [17]. The Ca2p energy spectrum which corresponded to P/i-PTFE/SCF/CaCO_3_ showed that 350.7 and 347.3eV belonged to CaO and CaCO_3_ [49]. These findings indicated that ZnO, TiO_2_, and CaCO_3_ nanoparticles underwent tribochemical reactions during the friction process, which facilitated the formation of intact lubricating film, to a certain extent. The formation of the lubricating film not only provided effective isolation between the composite and friction pair, but also enhanced the adhesion strength of transfer film, demonstrating excellent self-lubricating properties.

**Figure 11:**
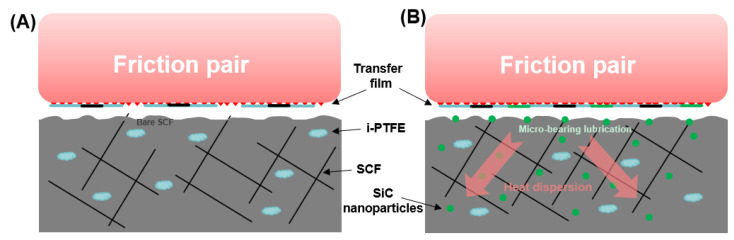
Schematic diagram of friction and wear behaviour of (**A**) P/i-PTFE/SCF and (**B**) P/i-PTFE/SCF/SiC.

Taking P/i-PTFE/SCF/SiC as an example, the schematic diagram of nanoparticle-containing P/i-PTFE/SCF composite during tribological measurements is displayed in Figure 11B. As shown in Figure 11A, the self-lubricating behaviour of P/i-PTFE/SCF composite primarily relied on the fluorinated transfer film originating from the transfer of i-PTFE and the carbon lubricant arising from SCF debris. However, the presence of randomly distributed SCF on the worn surface likely scraped off or destroyed transfer film which led to a higher wear loss. The samples which contained nanoparticles (here referred to SiC) not only utilised the lubricating effects of i-PTFE and SCF, but also enhanced the transfer film through the adhesion of SiC particles and friction-induced chemical reactions. In addition, SiC nanoparticles which were exposed to the friction interface played a role of “micro-bearing” effect. As a result, the sliding friction was transformed to rolling friction which reduced the direct impact of shearing action of the friction pair on the surface of the sample block. Furthermore, more intact thermally conductive pathways were likely constructed between the discretely distributed nanoparticles and SCFs. Under such circumstances, the friction-induced heat would be timely dissipated during the friction process which avoided the exacerbated abrasion of the substrate due to the accumulation of friction heat, thereby improving the friction and wear performance of corresponding composites.

A comparison of both the average μ and δ of PPS-based composites between this work and the reported literature is presented in Table 4. The results indicated that the friction and wear performance of P/i-PTFE/SCF/SiC composite outweighed those from the reported literature, suggesting that the use of ceramic inorganic nanoparticles such as SiC was instrumental in improving the tribological performance of PPS-based composites.

## 4. Conclusions

In this study, the influence of different types of inorganic nanoparticles (SiC, SiO_2_, ZnO, TiO_2_, or CaCO_3_) on the tribological and mechanical properties of PPS/i-PTFE/SCF composites was systematically investigated. The results showed that the introduction of solid inorganic nanoparticles significantly improved the bending strength and modulus. However, the inorganic nanoparticles-containing PPS/i-PTFE/SCF composites exhibited severe wear loss and temperature rise during the running-in stage. When the friction process was stabilised, the inorganic nanoparticles not only exhibited a ‘micro-bearing’ effect, but also promoted the formation of high-quality transfer films on the surface of the friction pair through enhanced adhesion and occurrence of friction-induced chemical reactions. Both factors were crucial to improving the tribological performance. Moreover, PPS/i-PTFE/SCF composites which were modified with inorganic nanoparticles exhibited adhesive wear, abrasive wear, and friction chemical reactions during the friction process. The PPS/i-PTFE/SCF/SiC composite demonstrated the best friction and wear performance among the studied systems which was due to its excellent thermal conductivity, resistance to external force, and the formation of high-performance lubricating transfer film. Specifically, the average friction coefficient and specific wear rate of PPS/i-PTFE/SCF/SiC composite reached 0.083 and 9.04 × 10^−6^ mm^3^/Nm, representing a reduction of 85.28% and 99.75% when compared with pure PPS, respectively. This work provided valuable insights for the fabrication of high-performance self-lubricating polymer composites that hold promising applications in high-end engineering sectors.

## Figures and Tables

**Table 1:** The compositions of inorganic nanoparticles-modified PPS/i-PTFE/SCF composites.

Sample ID	Composition (vol%)							
	PPS	i-PTFE	SCF	SiC	SiO_2_	ZnO	TiO_2_	CaCO_3_
P/i-PTFE/SCF	62.81	16.53	20.66	0	0	0	0	0
P/i-PTFE/SCF/SiC	57.75	15.19	19.06	8	0	0	0	0
P/i-PTFE/SCF/SiO_2_	57.75	15.19	19.06	0	8	0	0	0
P/i-PTFE/SCF/ZnO	57.75	15.19	19.06	0	0	8	0	0
P/i-PTFE/SCF/TiO_2_	57.75	15.19	19.06	0	0	0	8	0
P/i-PTFE/SCF/CaCO_3_	57.75	15.19	19.06	0	0	0	0	8

**Table 2:** The thermal analysis date of DSC measurements of PPS-based composites.

Sample ID	T_c_ (°C)	T_m_ (°C)	ΔH_m_ (J/g)	X_c_ (%)
P/i-PTFE/SCF	117.34	284.19	20.58	29.11
P/i-PTFE/SCF/SiC	114.06	283.28	17.52	27.95
P/i-PTFE/SCF/SiO_2_	115.52	282.38	20.30	28.60
P/i-PTFE/SCF/ZnO	116.23	282.23	14.98	23.12
P/i-PTFE/SCF/TiO_2_	117.23	283.04	16.03	26.51
P/i-PTFE/SCF/CaCO_3_	115.32	282.74	17.42	24.34

**Table 3:** The worn track parameters of specimens.

Sample	R_w_ (μm)	R_d_ (μm)
P/i-PTFE/SCF	2822.571	32.656
P/i-PTFE/SCF/SiC	5036.043	115.232
P/i-PTFE/SCF/SiO_2_	6233.177	179.507
P/i-PTFE/SCF/ZnO	6832.973	225.694
P/i-PTFE/SCF/TiO_2_	6950.581	266.067
P/i-PTFE/SCF/CaCO_3_	6527.195	235.156

**Table 4:** A comparison of the friction coefficient and wear rate of this work with literature values.

Sample ID	μ	δ (×10^−5^ mm^3^/Nm)	Reference
PPS/i-PTFE/SCF/SiC	0.083	0.904	This work
PPS/CuO/CF	0.221	4.36	[32]
PPS/PTFE/Al_2_O_3_	0.255	1.14	[50]
PPS/CNTs/SiC	0.231	5.01	[38]
PPS/PTFE/CF@GO	0.165	3.12	[51]
PPS/GR/CF	0.342	1.52	[52]
PPS/PTFE/PA	0.174	1.34	[53]
PPS/M-GO/M-CF	0.176	3.23	[54]
PPS/PFA/CF	0.137	1.72	[55]
PPS/TiO_2_/CF	0.512	2.12	[56]

## Data Availability

The original contributions presented in this study are included in the article. Further inquiries can be directed to the corresponding authors.
